# Transactions of the Buffalo Obstetrical Society

**Published:** 1885-01

**Authors:** 


### TRANSACTIONS OF THE BUFFALO OBSTETRICAL SOCIETY.

*Stated Meeting, Nov. 25, 1884.*

The President, Dr. W. W. Potter, in the Chair.

On invitation of the President, Dr. George E. Fell led a discussion on "The Value of the Hot Vaginal Douche in Pelvic Diseases."

Dr. Fell first called attention to the wide range of therapeutical application of heat and cold. By cold is meant a temperature below 26° C.; and by heat a temperature between 30° and 80° C. The physiological action of the warm bath—69° to 73° C.—causes, first, a soothing sensation with quickened pulse and respiration, and a more vigorous condition is temporarily produced through increased oxygenation. If prolonged, however, the stimulation is followed by depression, which is proportionate to the length of time of the immersion. In the hot bath—73° to 80° C.—these conditions follow each other with greater rapidity.

He then quoted from Dr. Ranney's paper, read before the New York Academy of Medicine, the present year, the author being a strong advocate of the beneficial effects of hot water, both as a local application and in its general use by imbibition. Used in the latter way it must be taken as hot as can be drunk, in order to avoid nausea. The functions of the abdominal organs are much improved by the prolonged use of this remedy; the nervous system, also, is favorably impressed, cases of hyperæmia and anæmia of the brain and spinal cord being greatly benefited. Dr. Ranney believes these favorable results are obtained through the influence produced upon the nerves of the stomach, and through them upon the solar plexus; *i. e.*, to a remote influence, upon the vaso-motor nerves or their centers.

Dr. Fell then proceeded to consider the benefits of hot water upon the female pelvic organs when diseased, calling attention to the fact that some of these results might be due to the proximal location of the hypogastric plexus to the uterus and vagina. The patient should be made to assume the dorsal recumbent posture, with the outlet of the vagina higher than its vault, to promote distention of the vaginal canal with the water. The bath should last, say, twenty minutes, and be given two or more times daily. Dr. Mundé recommends the gravity douche, while Dr. Emmet prefers the interrupted stream from a Davidson syringe. The temperature of the water has received much attention. It is best to commence with that of about 74 °C., and gradually increase the temperature until 80° to 85° C. is reached. Among the diseases in which this treatment has been found beneficial may be mentioned amenorrhœa, ovarian dysmenorrhœa, peri-uterine cellulitis, chronic cervical endometritis, metritis, vaginitis, and pruritus vulvæ. The purpose for which the injection is employed is somewhat of a guide to the requisite quantity; if for the toilet, a pint or a quart will suffice; if for disinfection, a larger quantity may be necessary, the flow to be continued until all odor is destroyed; if as an astringent, a smaller quantity will answer, but it should be retained for some time; if a hæmostatic effect is desired, the temperature should be as high as can be borne, the quantity large and the force considerable. A stimulant, or a tonic effect, can also be produced by varying the force, the amount and the temperature, as will readily occur to the intelligent observer. He concluded with the statement that the use of hot water, in the manner and for the purposes described, was first due to American gynecologists; but it has now become a recognized remedy of great superiority by physicians throughout the civilized world.

Dr. Charles G. Stockton considered hot water indispensable in gynecological practice, and, after using it several years, he was prepared to indorse all Dr. Fell had said in its favor. He believed that cold water, as popularly used, had been the source of much harm to women; but the effect produced, by alternating hot with cold water, might possibly prove beneficial in some conditions. Rapid changes of temperature in this way would increase the tone of the part to which it was applied, but great caution would be necessary in such applications. He regarded it of the first importance to properly place the patient while receiving the injection. His directions in this matter had been so often disobeyed, that he had resorted to various expedients to attain the desired end. Finally, he hit upon the following plan, which served a good purpose: Where the treatment would necessarily be prolonged, a small cot bed, costing about $2.00, is purchased by the patient, which is arranged with leather straps across its center by which the hips could be raised above the level of the rest of the body; a piece of oil cloth and a gravity syringe holding three gallons, arranged to exhaust itself in twenty minutes, would complete the armament. Upon this cot a patient can take her bath in comfort, without the danger of wetting or soiling her bed or linen. He also proposed, when heat and moisture were required for improving the nutrition of the pelvic organs, and hot water for any reason could not be made available, to introduce into the vagina a rubber tube crowned with sponge, the other end being attached to a steam atomizer. If dry heat was preferred, a gutta percha ball could be substituted for the sponge. He did not offer these suggestions as the result of experience; but, on theoretical grounds, he thought the plan, though crude, would warrant investigation, and believed it possessed considerable merit.

Dr. C. R. Jewett said that the first thing to determine was whether the disease which we propose treating by the method under discussion was acute or chronic, septic or idiopathic. While in chronic inflammations he believed in the use of hot water, in acute conditions more benefit could be derived from cold applications. In acute cellulitis, from any cause, he would apply cold by means of ice in rubber bags, placed over the abdomen. When the cellulitis was due to septic causes he would supplement this treatment with the hot vaginal bath. He believed that when cold was used early, it was capable of controlling any inflammation of the abdominal organs with reasonable certainty. It would produce all the effects which might be expected from the use of hot water, viz., contraction of the blood vessels, thereby diminishing the blood supply. Thereupon would follow a diminution of pain and a reduction of temperature. What more could be expected from hot water? In his opinion the benefits of cold had not been adequately recognized. For example, parametritis could be markedly relieved in from twelve to sixteen hours by its use, and general peritonitis thereby avoided. Certainly, a remedy that possessed such capabilities did not deserve neglect.

Dr. Jewett said he would not, however, be understood as disapproving the use of hot water as, in the main, described by the leader of the debate, in many of the chronic pelvic disorders of women. It promoted involution, and served to provoke absorption of plastic exudates in the pelvic connective tissue. It improved nutrition in many conditions where this was defective, and, on the whole, had been productive of much good. He then described the apparatus used in the New York hospitals for administering the hot vaginal bath, which consisted of a board placed over the bath tub, fitted with stirrups, and surrounded with a gutter to carry off the water. Upon this the patient could rest and take a bath from a gravity syringe quite comfortably. He had often seen a number of them reading at the same time, which rendered the treatment less irksome by diverting the mind.

Dr. P. W. Van Peyma remarked that, as the clinical effects of the hot vaginal douche had already been so well discussed, he would say a word as to the physiological effects of heat and cold. The latter produces its effects by causing contraction of the arterioles, and, in this way, acts as a styptic; heat, on the other hand, accomplishes the same results, but through its power to coagulate the blood. Hot water acts in chronic inflammations by increasing the migration of cells, thus removing effete matter. In the use of these remedies the length of time of the application is an important factor in the production of therapeutical results; with cold, the application must be continued for a long time in order to obtain benefit, while heat acts beneficially sooner. These physiological effects would indicate the cases, and the particular stages of any given case, in which the one or the other of these agents should be chosen.

Dr. M. Hartwig said he had not given much attention to this subject, but he coincided with Dr. Jewett's views of the application of ice in acute inflammations. He believed strongly in the use of *cold* in all acute and sub-acute inflammations within the pelvic cavity. He would apply it outwardly and inwardly; outwardly by means of pounded ice to the abdomen; inwardly, by introducing the same agent into the vagina. He believed all these inflammations—acute and sub-acute—to be septic in character; consequently cold was the proper remedy, because it retarded the morbid processes which were taking place—

nipped them in the bud, so to speak—if only applied early enough. On the other hand heat quickened these phenomena; wherefore it should be employed only after the inflammation became chronic. In chronic pelvic inflammations hot water was theoretically preferable, its main effects being to hasten the processes of repair in the direction which nature pointed out or desired. Practically in them, however, he had little experience with the remedy, and less faith. He then described an instrument by which, in giving the vaginal bath, the water could be thrown backwards and forwards as long as it kept warm, by means of which the clothing was thoroughly protected. When the water cooled it was thrown away, and the instrument re-charged.

Dr. R. L. Banta held that an acute inflammation could, many times, be aborted by the proper use of hot water, and in this respect he would differ with Dr. Hartwig; but he also believed that this could be done by passing cold water through a coil of tubing which entirely surrounded the locality of the inflammation, after the method of Thomas. He related two cases where he had introduced ice into the womb to control hemorrhage, and in both there resulted inflammation of the uterus, and death of the patients. He believed now that had he used hot water, he could have checked the inflammation after it developed, and saved his patients. This was his opinion after a greater familiarity with the use of the remedy, and he was now a thorough believer in it. He referred to Dr. Emmet as the father of the hot water treatment in the diseases of the female pelvic organs, and thought his teachings had been of untold benefit to woman.

Dr. S. Y. Howell said there was an apparatus made in Vienna (known as Leiter's coil) of malleable leaden pipe, which could be adapted to any part of the body, and was a valuable means of applying either hot or cold water treatment. He wished to take exception to the statement that all inflammations in the pelvic cavity were septic in origin. It seemed to him that an inflammation frequently arose into which sepsis did not enter as a cause. A woman has an attack of cellulitis after sitting on a cold surface, like stone or marble, which surely cannot be due to any septic influence. Given, a congestive state long enough continued, and inflammation would result in spite of any preceding septic condition. He quite approved of Dr. Stockton's cot, particularly the strap attachment to elevate the hips. He considered it one of the most effective ways a hot vaginal bath could be given. The syphon plan could be adapted to the greatest variety of cases, and in this respect must be considered the best. We must adapt our method, however, to the exigencies of each case, and not be tied to a set rule of procedure.

Dr. Thomas Lothrop thought the various points of the subject had already been pretty well covered by the discussion, though, in regard to the temperature of the water he had never found it expedient to resort to the higher degrees mentioned. About 105° F. was the average limit which he found useful; only rarely did he go beyond that point. One of the greatest difficulties to be met in private practice was the inconvenience which the treatment caused in some families. This seemed the principal ground of objection to it on the part of many patients, and he considered any plan good which combined efficiency with simplicity. Dr. Stockton's suggestion was a good one, but many patients could not, or would not, get the cot. If we try and carry out our plans with the material at hand we will often succeed, when, if too much be demanded nothing can be accomplished. The introduction of the hot water treatment had served to curtail the use of the speculum, and this he thought a great gain. In many cases, under intelligent advice and guidance, a woman could now cure herself, with only an occasional consultation with her physician. He believed the indiscriminate use of the speculum and the application of caustics, had caused many a woman to suffer needlessly, both physically and financially, who could have been relieved promptly and certainly with the hot water treatment properly and systematically carried out. Its capabilities for good, thus applied, were inestimable, and he regarded it as one of the most valuable agents we possess for the successful control of many diseases of women.

Dr. Wm. B. Hawkins said that attention to details was all that was necessary to ensure success with this treatment when it was indicated. If there was failure it was because of inattention to these, and not to the fault of the method. He was very particular with his patients in this regard and often found it necessary to administer the bath himself, so that they might fully understand just what was required.

Dr. C. C. Frederick believed that the great value of hot water was found in its effects upon the lesser pelvic inflammations. If used properly and in season, these could often be controlled and prevented from developing into more formidable conditions. He was frequently in the habit of instructing his patients to take the bath upon the floor, the hips resting upon a vessel which would raise them above the level of the trunk. If protected from cold he found this a simple and effective way of accomplishing the desired end.

The President remarked that the interest in this subject—and there had been no more important one before the society—centered chiefly in the almost universal applicability of hot water to the pelvic diseases of women. Were he limited, in the management of these diseases, to the choice of one remedial agent, he would, unhesitatingly, name hot water as the remedy, *par excellence*, having more capabilities for both the prevention and cure of the maladies in question, than any other known to him. While this might seem strong language, he believed that he was not over-lauding the remedy, and that in the statement he would be sustained by the members of the profession who have the largest experience in its use. Vaginal injections are of very early origin, but it is only lately that they have been properly applied to the treatment of disease. Dr. Emmet first taught the value of hot water at the Women's Hospital not more than twenty years ago, and to-day it is one of the standard therapeutic agents at the command of the gynecologist. It is probable, however, that its true value is not justly estimated by the profession as a whole. As the time had nearly arrived when the debate must close, he could only refer to two or three points pertaining to the subject. First, as to the position of the patient. The genu-pectoral posture was undoubtedly the best, because in it we have the important aid of gravity; it can, however, seldom be made available for this purpose, on account of the constrained attitude being illy borne for so long a time. The next most desirable is the dorsal position, which is the only really practicable posture for this treatment, and which has been fully discoursed upon. The crouching position, though quite common, is really worse than useless for any purposes of treatment, and need not claim further consideration. Second, as to the instrument. The Davidson's syringe serves as the type of the class furnishing the interrupted current, and this current on many accounts is preferable. The Foster's douche apparatus he had found the most convenient of any instrument of this class. The fountain syringe illustrates the class, deriving its force from gravity, of which there are many useful modifications. The gravity current is made to serve a good purpose where the patient must administer the injection herself, but when she could command an assistant he preferred the Foster's syringe. Whatever instrument is used the nozzle should be made of hard rubber without a central aperture, the reasons for which are obvious. Third, as to temperature. A safe guide was to adapt the degree of heat to the comfort of the patient, always having in view the purposes of the application; for it will be found that now a lower, and again a higher temperature, will be borne. In many diseases the highest temperature which could be tolerated was the most beneficial. The duration and frequency of the lavements must be determined by the necessities of each case.

He called attention to the fact that hot water *per rectum* could be made to serve a good purpose in many cases of pelvic disease. His attention had been first directed to this method of treatment by a paper by Dr. Chadwick, published in the transactions of the American Gynecological Society, Vol. V, and he had since employed it with satisfaction in a number of instances. He commended it to the society as a supplementary resource of great value in many cases.

Dr. Fell, in closing the discussion, presented a general review of the subject, calling particular attention to the fact that the action of heat applied in this way, exerted its beneficial effects through its action on the nervous system. He also pointed out that the degree of temperature mentioned, as employed by some of the members, was much too low to derive the greatest benefits from the measure.

